# Prenatal phenotype analysis and mutation identification of a fetus with meckel gruber syndrome

**DOI:** 10.3389/fgene.2022.982127

**Published:** 2022-08-19

**Authors:** Laura Moreno-Leon, Marco A. Quezada-Ramirez, Evan Bilsbury, Courtney Kiss, Andrea Guerin, Hemant Khanna

**Affiliations:** ^1^ Department of Ophthalmology and Visual Sciences, UMass Chan Medical School, Worcester, MA, United States; ^2^ Kingston Health Sciences Centre, Queen’s Medical School, Kingston, ON, Canada

**Keywords:** cilia, ciliopathies, *RPGRIP1L*, meckel-gruber syndrome, ciliary defects

## Abstract

Ciliopathies are a class of inherited severe human disorders that occur due to defective formation or function of cilia. The *RPGRIP1L* (retinitis pigmentosa GTPase regulator-interacting protein1-like) gene encodes for a ciliary protein involved in regulating cilia formation and function. Mutations in *RPGRIP1L* cause ciliopathies associated with severe embryonic defects, such as Meckel-Gruber Syndrome (MKS). Here we report *RPGRIP1L* mutation analysis in a family diagnosed with MKS. The clinical manifestations of the fetus included thoraco-lumbar open neural tube defect with associated Chiari type II malformation and hydrocephalus, bilateral club feet, and single right kidney/ureter. Analysis of the parental DNA samples revealed that the father carried a previously reported mutation R1236C/+ whereas the mother had a novel splice site mutation IVS6+1 G > A/+ in *RPGRIP1L*. The splice site mutation resulted in the exclusion of in-frame exon 6 of *RPGRIP1L* (RPGRIP1L-∆Ex6) but expressed a stable protein in fibroblasts derived from the parents’ skin biopsies. The GFP-RPGRIP1L-∆Ex6 mutant protein exhibited relatively reduced ciliary localization in transiently-transfected cultured RPE-1 cells. Taken together, this study identifies a novel RPGRIP1L variant RPGRIP1L-∆Ex6, which in combination with RPGRIP1L-R1236C is associated with MKS. We also suggest that the deletion of exon 6 of RPGRIP1L leads to reduced ciliary localization of RPGRIP1L, indicating a plausible mechanism of associated disease.

## Introduction

Cilia are extracellular membrane extensions, which function primarily as sensors to mediate communication between the cell and the extracellular environment (Satir and Christensen, 2007). Disruptions to cilia or cilia anchoring structures leads to a group of pleiotropic genetic disorders called ciliopathies (Hildebrandt et al., 2011). These disorders are characterized by extreme genetic heterogeneity and phenotypic variety. Many of the phenotypic characteristics of these disorders are caused by the disruption of normal cilia function in the process of embryonic development (Eggenschwiler and Anderson, 2007). Despite the identification of numerous genes involved in ciliopathies the role of these genes in disease is still being delineated.

Meckel syndrome (MKS) and Joubert syndrome (JBTS) are severe, recessive neurodevelopmental disorders caused by mutations in a variety of genes leading to defective primary cilia which disrupts the process of embryonic development (Brancati et al., 2010; Parelkar et al., 2013). Although they are clinically distinct syndromes, there is large genetic/allelic overlap between MKS and JBTS, as well as between these disorders and other ciliopathies such as Bardet-Biedl syndrome (BBS) (Baker and Beales, 2009; Szymanska et al., 2014). The most common clinical features of these disorders are rod-cone dystrophy, post axial polydactyly, neural tube defects (NTD) and renal/genitourinary malformations. The most severe, MKS, is the most common form of syndromic NTD and is neonatally lethal (Logan et al., 2011).

Mutations in 13 genes can account for 75% of all cases of MKS. Among these genes, the most strongly associated with MKS include *CEP290*, *MKS1* and *RPGRIP1L*. Retinitis pigmentosa GTPase regulator-interacting protein-1 like (*RPGRIP1L*), also known as *MKS-5* and *NPHP8*, is a ciliary protein that localizes to the base of the cilium and interacts with retinitis pigmentosa GTPase regulator (*RPGR*) (Arts et al., 2007; Khanna et al., 2009). Essential to the function of *RPGRIP1L* is its ability to localize to the base of the cilium where it controls ciliary gating by maintaining the amount of *CEP290* at the transition zone (TZ) (Wiegering et al., 2021). Disruption of these properties can lead to phenotypes encompassed by MKS and JBTS (Delous et al., 2007; Chen et al., 2011).

Here, we characterize a Canadian family of U.K (paternal) and presumed European descent (maternal) with a history of MKS carrying compound heterozygous variants in *RPGRIP1L*. Identification and characterization of these variations allows for an increased understanding of the pathophysiological processes leading to ciliary dysfunction in MKS and retinal degeneration.

## Results

### Clinical examination

Routine fetal anatomy ultrasound at 19 weeks revealed a large neural tube defect involving the thoracic spine, ventriculomegaly (measuring 11–15 mm), Chiari malformation, bilateral club feet and two vessel cord/single umbilical artery. Fetal kidneys, cavum septi pellcuidi and cisterna magna were unable to be visualized. Amniocentesis was pursued for genetic testing by way of QF-PCR for chromosomes 13, 18, 21 (normal) and sex chromosomes (consistent with a male fetus). Fetal chromosomal microarray analysis did not reveal any clinically significant copy number changes. Fetal whole exome sequencing (including submission of parental samples to perform trio analysis) revealed a paternally inherited *RPGRIP1L* variant c.706C > T/p.R1236C and a maternally inherited *RPGRIP1L* variant c.776 + 1G > A/IVS6 + 1G > A. No additional variants were reported including no American College of Medical Genetics and Genomics secondary findings. The pregnancy was terminated at 20 weeks 3 days gestation due to the presence of multiple fetal anomalies.

External physical examination of the fetus by geneticist was consistent with an open neural tube defect; mild 5th finger clinodactyly was also present. Fetal autopsy showed thoracolumbar region open neural tube defect, cerebellar tonsillar herniation/Chiari type II malformation, bilateral club feet, imperforate anus, single right kidney/ureter, single or fused midline adrenal gland and two umbilical vessels. Radiologial skeletal survey showed multiple formation/segmentation defects evident throughout the spine with widening/dysraphic change apparent within the lower thoracic lumbar and sacral regions. Neuropathological examination showed extensive thoraco-lumbar open neural tube defect with associated Chiari type II malformation and hydrocephalus. We specifically asked about the possibility of molar tooth sign given that this is often a finding in Joubert syndrome and related disorders; neuropathology was unable to confirm or rule out molar tooth sign upon further examination.

### Genomic analysis of the *RPGRIP1L* gene in the family

**FIGURE 1:**
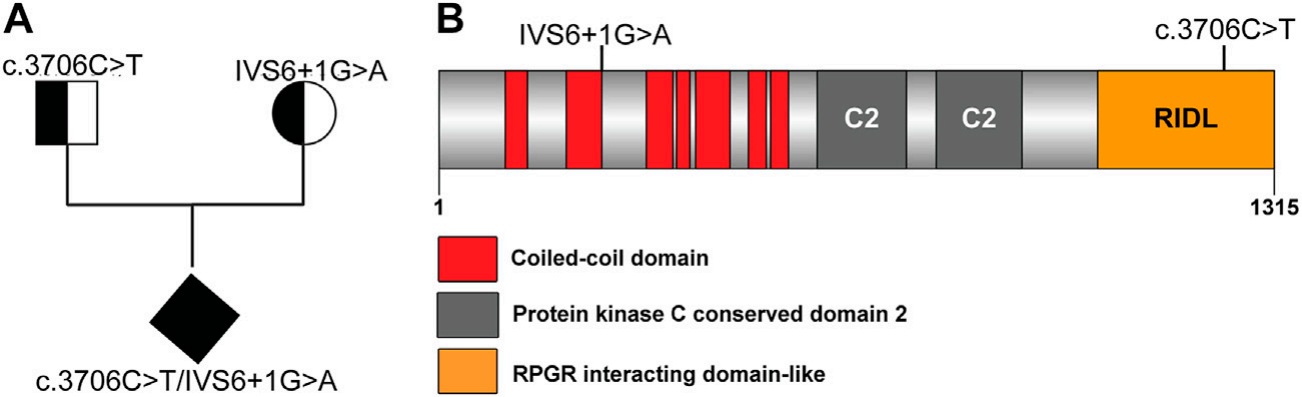
Novel *RPGRIP1L* mutation identified in an unborn fetus. **(A)** Pedigree of the family carrying *RPGRIP1L* mutations. **(B)** Schematic representation or the *RPGRIP1L* mutations reported in *RPGRIP1L* patients.

Whole genome sequence analysis of the miscarried fetus identified two potentially disease-associated variants in *RPGRIP1L* (Supplementary Figure S1). The asymptomatic mother carried a heterozygous variant *RPGRIP1L* (NM_015272.2):IVS6+1G > A and the asymptomatic father was heterozygous for *RPGRIP1L* (NM_015272.2):c.3706C > T (R1236C). The fetus, on the other hand had compound heterozygous mutations possessing both variants (Figure 1A). While IVS6+1G > A is located at the donor splice-site between exon 6 (encoding a coiled-coil domain) and intron 6, c.3706C > T (R1236C) is located within the RPGR-interacting domain-like region of *RPGRIP1L* (Figure 1B).

### *RPGRIP1L* expression analysis in fibroblasts

**FIGURE 2:**
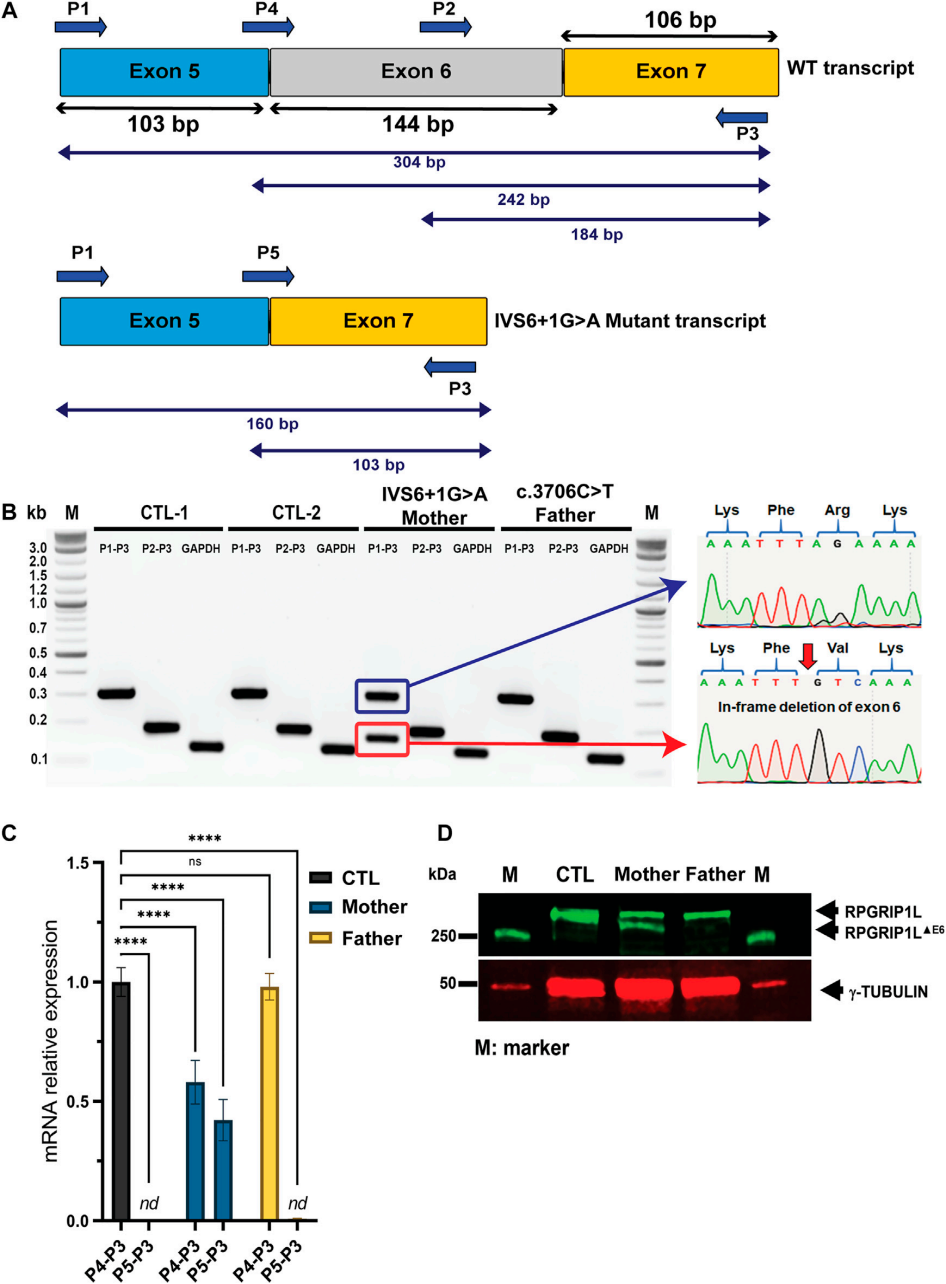
Molecular characterization of the novel *RPGRIP1L* IVG6+1G > A mutation in a Canadian family with frequent birth miscarriage. **(A)** Structure of the *RPGRIP1L* gene flanking the IVG6+1G > A mutation with exons size drawn to scale and predicted alternative splicing. Lower panel illustrates the anticipated exon 6 skipping. **(B)** RT-PCR analysis using the indicated primer combinations was performed using fibroblasts derived from the mother and father samples as well as two unrelated controls (CTL-1 and CTL-2) A shorter transcript (∼160 bp; red box) was detected in the mother carrying the splice site variant. Sanger sequencing of the aberrant product confirmed the exclusion of exon 6. **(C)** Quantification of the exon 6-skipped *RPGRIP1L* isoform was performed by qRT-PCR analysis. The results are analyzed from three independent experiments. *nd*, *non detectable*. **(D)** Immunoblotting using *RPGRIP1L* antibody (green) and *γ*-tubulin (red) showing skipping of exon 6 (RPGRIP1L-ΔEx6) leads to the expression of an aberrant shorter polypeptide in the fibroblasts derived from the mother.

Point mutations in the 5′ donor (GT) or 3′ acceptor (AG) splice-sites can lead to either exon skipping or intron retention (Baralle, 2005). IVS6+1G > A is located at the exon-intron boundary between exon 6 and intron 6. Splice site prediction analysis revealed that the IVS6+1G > A mutation leads to the loss of the donor splice-site sequence motif by replacing the Guanine residue in the GT consensus sequence with an Adenosine residue (Supplementary Figure S2A). We hypothesized that loss of the 5′ donor splice-site at the 5′-end of intron 6 would lead to skipping of exon 6 (Figure 2A). To test this hypothesis, we generated fibroblasts from skin biopsy specimens of the IVS6+1G > A/+ (mother) as well as control fibroblasts and examined the expression of *RPGRIP1L* mRNA using primer combinations encompassing the splice-site region (Supplementary Table S1 and Figure 2A). RT-PCR analysis using primer pair P1-P3 showed the expression of a shorter PCR product (160 bp) in addition to the expected WT–RPGRIP1L (wild type; ∼304 bp) product. Sequence analysis of the shorter product revealed a deletion of exon 6 (RPGRIP1L-∆Ex 6) (Figure 2B). No change in the PCR profile of *RPGRIP1L* was observed in the R1236C/+ parent fibroblasts.

We next performed quantitative RT—PCR (qRT-PCR) analysis to ask whether the identified variants affect the mRNA levels of *RPGRIP1L* in parents’ fibroblasts (Figure 2C). As predicted, mRNA was undetectable for the P5-P3 primer combination (encompassing the mutant transcript without exon 6) in the control and R1236C/+ fibroblasts. The wild-type and the mutant transcripts with primer pairs P5-P3 (mutant transcript) and P4-P3 (wild-type) were detected at 50% levels in the IVS6+1G > A/+ fibroblasts (p ≤ 0.0001).

We then performed immunoblotting of protein extracts of parents’ fibroblasts using an anti-*RPGRIP1L* antibody (Figure 2D). Bands corresponding to *RPGRIP1L*-∆Ex6 and *RPGRIP1L* were observed in the IVS6+1G > A/+ fibroblasts indicating the production of an aberrant shorter polypeptide. A single band, corresponding to the full-length *RPGRIP1L* protein was observed in the R1236C/+ fibroblasts. The specificity of the *RPGRIP1L* antibody was validated by immunoblotting using the HEK293 cells transiently transfected with plasmids encoding the GFP-fused *RPGRIP1L* WT and mutant proteins (Supplementary Figures S2B,C).

### Ciliary localization of *RPGRIP1L*

**FIGURE 3:**
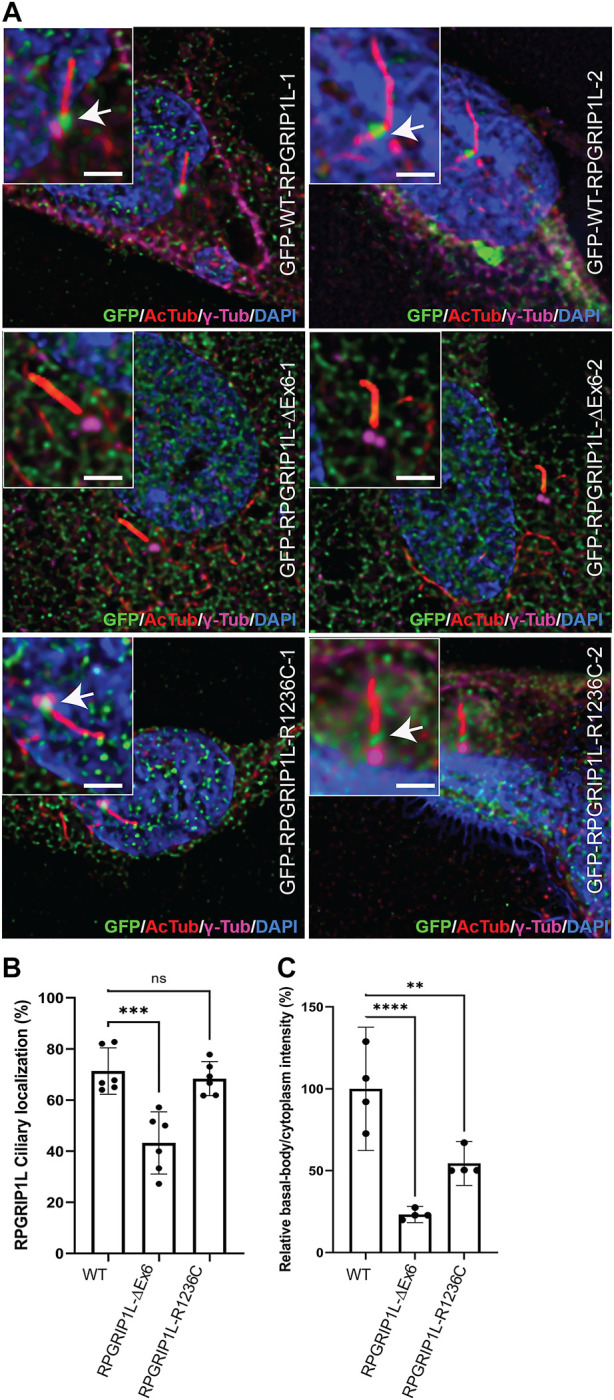
Ciliary distribution of the RPGRIP1L-WT-GFP, RPGRIP1L-ΔEx6-GFP and RPGRIP1L-R1236C-GFP in hTERT-RPE1 cells. **(A)** Immunostaining of the ciliary markers acetylated-tubulin (red) and *γ*-tubulin (magenta) showing the absence (RPGRIP1L-ΔEx6-GFP, green) or reduction (RPGRIP1L-R1236C-GFP, green) of the mutant *RPGRIP1L* localization at the cilia. Insets show the localization of *RPGRIP1L* to the proximal transition zone region of the cilium. Scale: 2 μm **(B)** Quantification of the ciliated cells with RPGRIP1 localization either to the basal body or to the transition zone showed significant reduction of RPGRIP1L-ΔEx6 at the cilia. **(C)** Quantification of *RPGRIP1L* intensity at the basal body compared to the cytoplasm showing a reduction of *RPGRIP1L* ciliary localization of both RPGRIP1L-ΔEx6 and RPGRIP1L-R1236C mutants.

*RPGRIP1L* has previously been shown to localize to the BB of cilia and function as a ciliary gatekeeper allowing only certain proteins to cross the TZ (Lin et al., 2018; Wiegering et al., 2021). To test if the observed variations in *RPGRIP1L* alter its ability to localize to the BB, we transiently transfected hTERT-RPE1 cells with plasmid DNA encoding GFP-fused *RPGRIP1L*-WT, RPGRIP1L-∆Ex6 or RPGRIP1L-R1236C followed by cilia growth and immunofluorescent staining using acetylated tubulin (AcTub; cilia marker), γ-tubulin (γ-Tub; BB marker), and GFP antibodies (Figure 3A). Consistent with previous reports (Arts et al., 2007), RPGRIP1L-WT and RPGRIP1L-R1236C co-localized with γ-Tub to the BB of the cilia in ∼70% of the cells (arrows in Figure 3A). In contrast, BB localization of RPGRIP1L-∆Ex6 was detectable only in ∼40% of the cells (Figure 3B; *p* ≤ 0.001). Quantification of the BB/cytoplasm intensity ratio of *RPGRIP1L* showed ∼80% reduction in the localization of RPGRIP1L-∆Ex6 at the BB (p ≤ 0.0001) (Figure 3C). A statistically significant reduction (p ≤ 0.01) in relative intensity ratio was also observed for RPGRIP1L-R1236C compared to RPGRIP1L-WT (Figure 3C).

## Discussion

Several of the known ciliopathy-causing genes either contribute to a recessive form of the disease (such as in MKS) or modulate the penetrance and expressivity of other causative genes. The significant pleiotropy of *RPGRIP1L* is reflected by the multiple names (*MKS5*, *NPHP8*, *JBTS7*) by which it is referred to when involved in distinct disease processes of varying severity (Andreu-Cervera et al., 2021). Even within the spectrum of MKS, pleiotropy exists between different types of mutations; for example, while homozygous truncating mutations of *RPGRIP1L* typically produce MKS it has been shown that the presence of at least one missense mutation can modify the disease severity and lead to less severe and later onset disease (Reiter and Leroux, 2017). Homozygous missense mutations, frameshift, and splice-site mutations in *RPGRIP1L* have been reported in individuals with JBTS, another severe ciliopathy (Devuyst and Arnould, 2008). Additionally, polymorphic variation in *RPGRIP1L* has been shown to be a modifier of retinal degeneration in ciliopathies (Khanna et al., 2009). Understanding the molecular basis for the varying severity of these mutations is essential for the accurate prediction of phenotypes *via* genetic screenings in the future.

Exon 6 of *RPGRIP1L* encodes a coiled-coil domain. Coiled-coil domains have previously been shown to act as “tags” for oligomerization, notably, of the BBS2 and BBS7 subunits of the BBSome, which is an octameric protein complex involved in regulating protein trafficking at the basal body (BB) of primary cilia (Chou et al., 2019). The BBSome is thusly named because 7 of the eight proteins involved are known causes of Bardet-Biedl syndrome (BBS), another ciliopathy characterized by rod-cone dystrophy, polydactyly, and kidney dysfunction. In addition to this, the coiled-coil domain of exon 6 in *RPGRIP1L* has been shown to be necessary for the proper localization of CEP290 to the transition zone (Li et al., 2016).

Our results show that exon 6 is necessary for proper localization of *RPGRIP1L* to the TZ. There are 7 coiled-coil domains in the protein all of which may engage in oligomerization at the TZ. In *C. elegans*, it has been shown that MKS-5 (mammalian RPGRIP1L/RPGRIP1 orthologue) is crucial for the assembly of a ciliary transition zone and is required for the correct localization of all known *C. elegans* transition zone proteins (Jensen et al., 2015). Jensen et al. were able to demonstrate that the coiled-coil domains of *C. elegans* MKS-5 were, in fact, sufficient for transition zone localization of the protein through truncation studies. This suggests a similar role of the coiled-coil domain of exon 6 in human *RPGRIP1L*.

MKS displays an autosomal recessive (AR) inheritance pattern and parents are usually not aware that they are carriers until after they have an affected child. Once pathogenic variants have been identified in a family, it is possible to prenatally diagnose the disorder using chorionic villus sampling or amniocentesis (Watson et al., 2016). Alternatively, some families may proceed with assisted reproductive technologies such as *in vitro* fertilization with pre-implantation genetic testing for monogenic disease (IVF with PGT-M) to avoid having an affected pregnancy. However, the identification of pathogenic variants is often hindered by an incomplete understanding of the role of different alleles within previously identified causative MKS genes. Currently, the genetic and molecular mechanisms which lead to MKS are known in only ∼60% of cases (Szymanska et al., 2012). It has been documented that in some cases, multiple allelism at a single locus or within a single gene can account for some of this phenotypic variability (Chang et al., 2006; Chen, 2007). Recently novel compound variants have been reported in CEP290 (Peng et al., 1935), and aggregating results regarding mutations in *MKS1* have resulted in a proposed genotype-phenotype correlation linking the severity of mutations and the domains affected to diagnoses of either MKS, JBTS or BBS (Leitch et al., 2008; Luo et al., 2020; Lin et al., 2022). Further studies are needed to not only understand the genotype-phenotype correlation in ciliopathies but also formulate efficient genetic testing so that counseling can be provided to families predisposed to developing debilitating ciliopathies. Based on these results, the family was offered assisted reproductive technologies for future pregnancies. They are currently pursuing *in vitro* fertilization with egg donor. The family was counselled regarding all options, including PGT-M.

## Methods

### Study approval

The skin biopsies were obtained from *RPGRIP1L* patients and previously published unaffected individuals after written informed consent. Written consent was obtained from the family for publication. All procedures followed the Declaration of Helsinki and approved by the Institutional Review Board of the University of Toronto and UMass Chan Medical School.

### Plasmid DNA constructs

Plasmids encoding human WT or mutated *RPGRIP1L*s were obtained with cDNA synthesized from parental skin biopsies using SuperScript™ First-Strand Synthesis System for RT-PCR (Life Technologies). The *RPGRIP1L* coding region was amplified with PrimerSTAR GXL polymerase (Takara) and directly cloned into pMiniT vector (New England Biolabs). The thermal cycle setting was: 98°C for 2 min (activation), 98°C for 10 s and 68°C for 6 min (annealing/extension), for a total 40 cycles. Subsequenlty, *RPGRIP1L* was subcloned into pEGFP-C1 to express N-terminal GFP-tagged proteins under the control of CMV promoter. Primer details are provided in Supplementary Table S1.

### Fibroblast isolation, cell culture and transient transfection

The skin biopsies were dissociated as previously described to obtain primary fibroblasts (Shimada et al., 2017; Moreno-Leon et al., 2020). Briefly, biopsies were washed in iodine followed by three washes in phosphate-buffered saline (Invitrogen). The sample was minced with a sharp scalpel in a culture plate and incubated with Dulbecco’s-modified Eagle’s medium (DMEM)/F12 (Gibco) supplemented with 20% fetal bovine serum (FBS) (Sigma-Aldrich, Saint-Louis, MO, United States), penicillin and streptomycin at 37°C in a humidified 5% CO2 incubator. Ten days after harvesting, the skin was removed, and fibroblasts were passaged for storage and analysis.

Dissociated primary fibroblasts and hTERT-RPE1 cells (ATCC, CRL-4000) were grown in DMEM/F12 media (Gibco) while HEK293 cells were grown in DMEM media (Gibco), 1% Sodium Pyruvate. All cells were supplemented with 10% FBS (Sigma-Aldrich, Saint-Louis, MO, United States) and grown at 37°C and 5% CO_2_ incubator. All analyses were done in the fibroblasts at the same passage stages.

Plasmid DNA transfections were performed using Lipofectamine 2000 (Invitrogen) in HEK293 cells and FuGENE 6 (Promega) in hTERT-RPE1 cells at a 3:1 Transfection Reagent: DNA ratio according to the manufacturer’s protocol.

### RNA extraction and quantitative reverse transcriptase-polymerase chain reaction analysis

Total RNAs were isolated from primary fibroblasts with QIAzol Lysis Reagent (Qiagen) and precipitated according to the manufacturer’s instructions. Reverse transcription was performed with 1 μg of total RNAs using the SuperScript™ First-Strand Synthesis System for RT-PCR (Invitrogen). The resulting cDNA was used to perform DNA amplification using PrimeStar GXL DNA polymerase (Takara) according to manufacturer’s instruction. Alternative splicing defects were analyzed by agarose gel electrophoresis. Gene expression analysis was performed using the BioRad CFX96 qPCR instrument and Power SYBR Green PCR Master Mix (Thermo Fisher Scientific). All RT-PCR and RT-qPCR primers are described in Supplementary Table S1.

### Immunoblotting

The cells were lyzed on ice in Pierce RIPA Buffer (Thermo Scientific) containing Halt™ Protease Inhibitor Cocktail (100X, Thermo Fisher). Lysates were sonicated and centrifugated at 12 000 g for 15 min. Supernatants were quantified and 20 μg of total protein lysate was incubated for 5 min at 95°C with Laemmli sample buffer. Proteins were separated on a 4–20% Mini-PROTEAN® TGX™ Precast Protein Gel (BioRad) and transferred to a nitrocellulose membrane (BioRad). The blots were processed using LI-COR western blotting instructions and blocking, and primary and secondary antibodies solutions were prepared in Intercept Blocking buffer (LI-COR). Primary antibodies were incubated overnight at 4°C and secondary antibodies were incubated at room temperature for 2 h (Supplementary Table S2 for antibodies details and dilution information). Protein expression was detected using LI-COR Odyssey Fc detection system.

### Immunofluorescence, microscopy and co-localization analysis

The primary fibroblasts and hTERT-RPE1 cells were plated at 90% confluency on coverslips (AmScope CS-R18-100). Primary fibroblasts were serum-starved for 48 h, 24 h after plating. hTERT-RPE1 cells were serum-starved 24 h post-transfection for 24 h. Cells were fixed in 4% paraformaldehyde/1X Dulbecco’s phosphate-buffered saline (DPBS; potassium chloride 0.2 g/L, potassium dihydrogen phosphate 0.2 g/L, sodium chloride 8 g/L and disodium hydrogen phosphate 1.15 g/L) for 10 min, washed three times in DPBS and blocked in DPBS containing 5% normal goat serum and 0.5% Triton X-100 for 1 h. Primary antibody solution was prepared in blocking solution and incubated overnight at 4°C. After washing in DPBS, cells were incubated in blocking solution containing Alexa-488-conjugated and Alexa-546-conjugated (Invitrogen) for 1 h. Finally, cells were washed in DPBS and incubated for 5 min with DAPI for nuclear staining. Coverslips were mounted with Antifade Mounting Media (Vectashield) and all images were visualized over ×63 objective using a Leica DM6 Thunder microscope with a 16-bit monochrome camera.

The IMARIS platform was used to process the two channel intensities simultaneously and to measure the degree of overlap of the two channels. After setting intensity threshold, the basal body marker γ-tubulin was used to define a region of interest and a co-localization channel was built on different z-stacks to obtain the percentage of co-localized signal and intensity. When needed, adjacent optical sections were merged in the *z*-axis to form a projected image (ImageJ). Signal intensity and Co-localization values were plotted using the GraphPad Prism software.

### Statistics

All data are presented as means ± standard derivation from the mean. Two groups comparison was analyzed by Student’s t-tests using the Prism GraphPad software. Multiple groups comparison was performed with one-way analysis of variance. Differences between groups were considered statistically significant if *p* < 0.05. The statistical significance is denoted with asterisks (*p < 0.05; **p < 0.01; ***p < 0.005; ****p < 0.001).

## Data Availability

The original contributions presented in the study are included in the article/Supplementary Material, further inquiries can be directed to the corresponding author.
