# Supplementary material for: Prenatal phenotype analysis and mutation identification of a fetus with meckel gruber syndrome
Source: Front Genet. 2022 Aug 19;13:982127. doi: 10.3389/fgene.2022.982127 (PMC9437271; doi:10.3389/fgene.2022.982127)
Supplement: Supplementary file 1 [file Table1.pdf]

Table S1. RT-PCR and RT-qPCR primers

Name	Localization	Sequence	Application
P1	Exon 5	AGCAGAAACTCCACATCCCA	RT-PCR
P2	Exon 6	AGCACTTGGCTGAGATCCTG	RT-PCR
P3	Exon 7	CCTTCCATTGCTGAAAGAGCA	RT-PCR
P4	Exon 5/6	GAAATAAGAAATTTAGAAAACGTTATTCAGTCAC	RT-qPCR
P5	Exon 5/7	GAGGAGAAATAAGAAATTTGTCAAATATTCGGGA	RT-qPCR
Fwd-EcoRI	NM_015272.5	GCGTAGAATTCCATGTCTGGTCCAACTGATGAGA	Cloning
Rev-BamHI	NM_015272.5	TATAGGATCCTCAAGCCTCCAAGTCATCTCTGT3	Cloning
