# Supplementary material for: Prenatal phenotype analysis and mutation identification of a fetus with meckel gruber syndrome
Source: Front Genet. 2022 Aug 19;13:982127. doi: 10.3389/fgene.2022.982127 (PMC9437271; doi:10.3389/fgene.2022.982127)
Supplement: Supplementary file 2 [file Table2.pdf]

Table S2. Antibodies and dilution used

Name	Product information	Application	Dilution
Chicken anti-GFP	Abcam, # ab13970	Immunoblotting	1:1000
		Immunofluorescence	1:200
Rabbit anti-RPGRIP1L	Invitrogen, # PA5-100049	Immunoblotting	1:1000
Mouse anti-Gamma-Tubulin	Sigma Millipore, #T6557	Immunoblotting	1:2000
		Immunofluorescence	1:500
Goat anti-Mouse 680RD	Licor, # 926-68070	Immunoblotting	1:2000
Goat anti-Rabbit 800CW	Licor, # 926-32211	Immunoblotting	1:2000
Goat anti-Chicken-488	Invitrogen, # A32931	Immunofluorescence	1:500
Goat anti-Rabbit-546	Invitrogen, # A-11008	Immunofluorescence	1:500
Goat anti-Mouse-633	Invitrogen, # A-21052	Immunofluorescence	1:500
DAPI	Invitrogen, # D1306	Immunofluorescence	1:1000
