# Supplementary figures and images for: Prenatal phenotype analysis and mutation identification of a fetus with meckel gruber syndrome
Source: Front Genet. 2022 Aug 19;13:982127. doi: 10.3389/fgene.2022.982127 (PMC9437271; doi:10.3389/fgene.2022.982127)

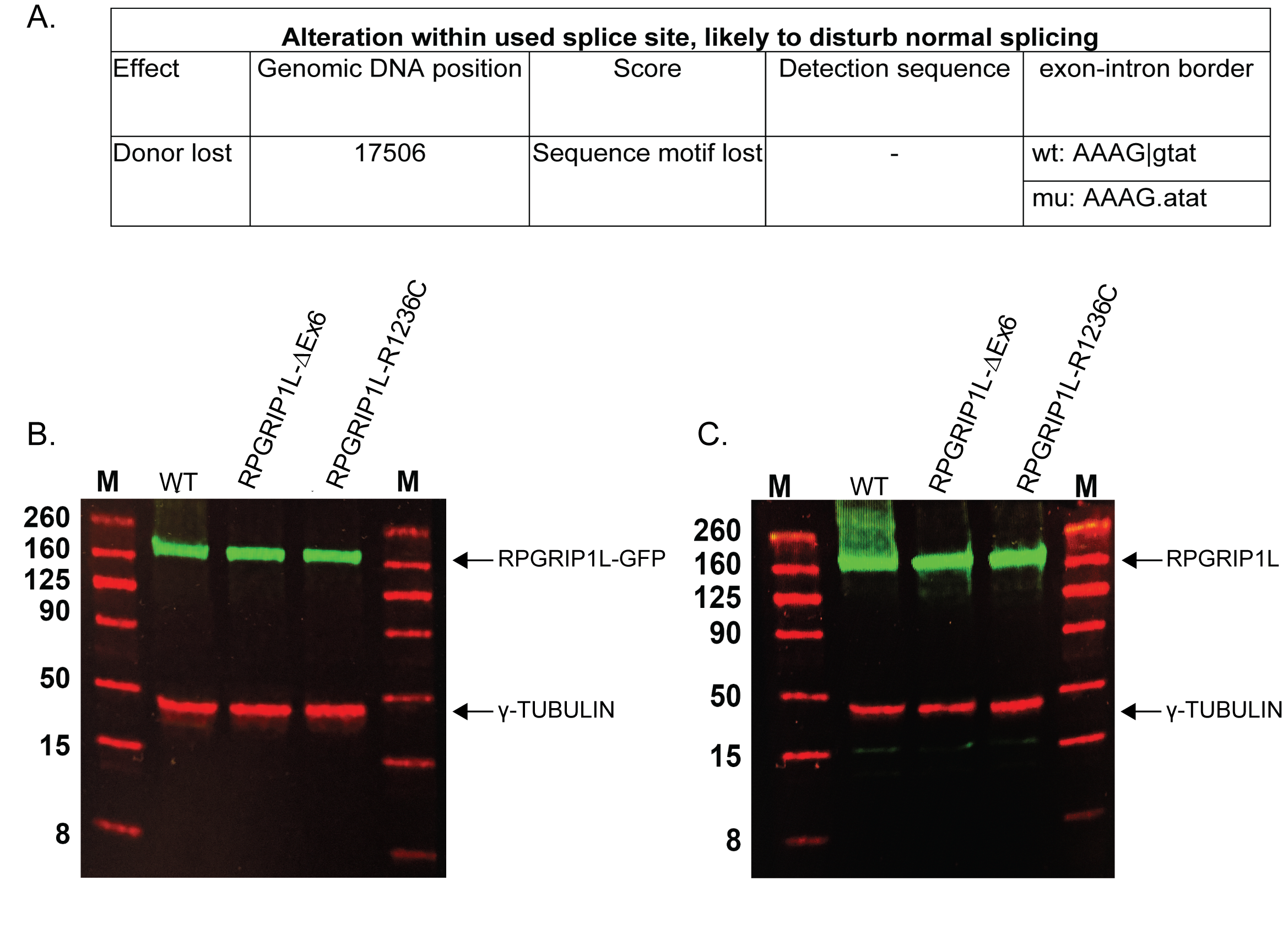

Supplement: Supplementary file 3 [file Image2.TIF]

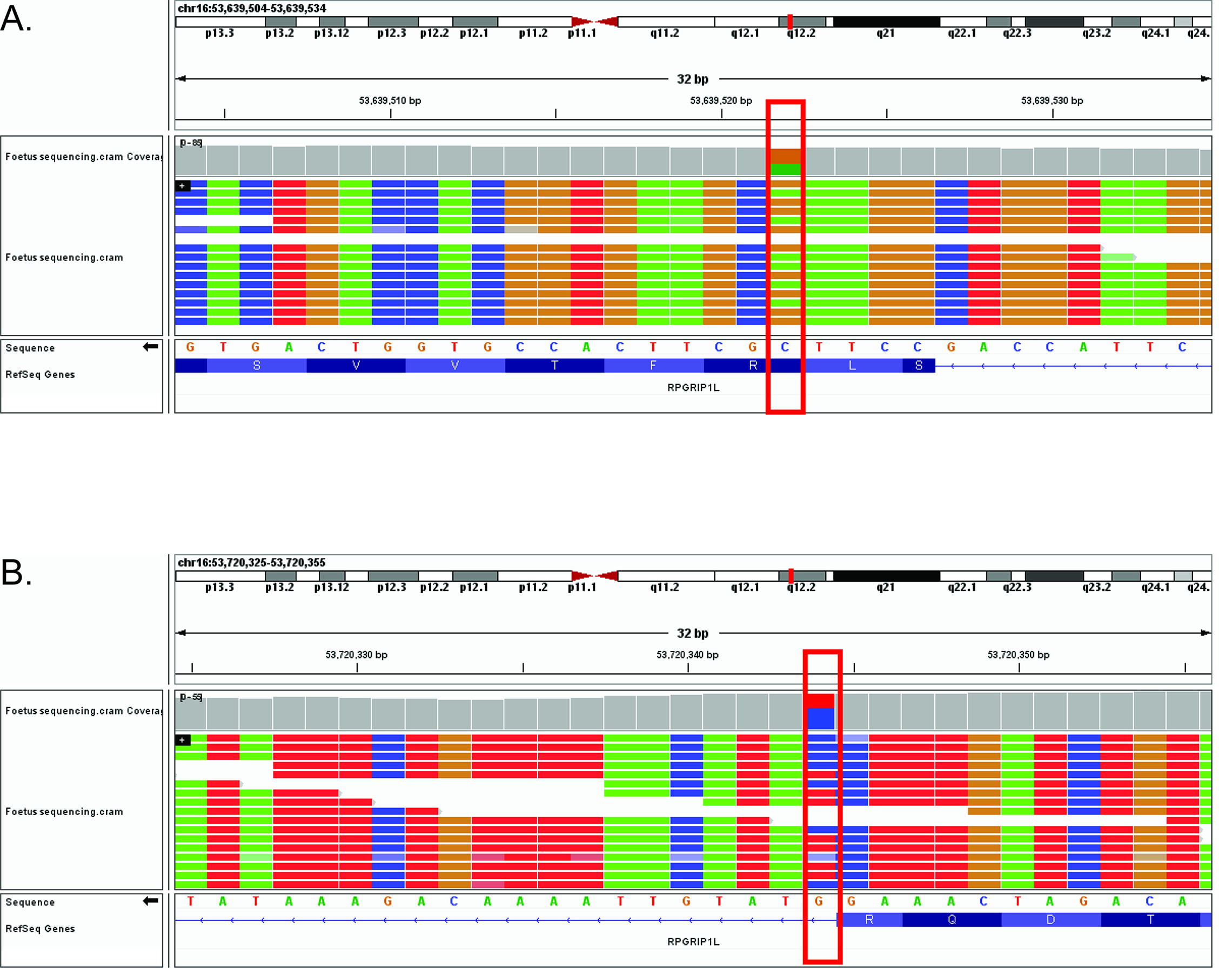

Supplement: Supplementary file 4 [file Image1.TIF]
